# Association of Pertussis Toxin with Severe Pertussis Disease

**DOI:** 10.3390/toxins11070373

**Published:** 2019-06-27

**Authors:** Karen Scanlon, Ciaran Skerry, Nicholas Carbonetti

**Affiliations:** Department of Microbiology & Immunology, University of Maryland School of Medicine, Baltimore, MD 21201, USA

**Keywords:** Pertussis toxin, bacterial infection, respiratory disease, leukocytosis, pulmonary hypertension, immunomodulation, inflammation, cough

## Abstract

Pertussis, caused by respiratory tract infection with the bacterial pathogen *Bordetella pertussis*, has long been considered to be a toxin-mediated disease. Bacteria adhere and multiply extracellularly in the airways and release several toxins, which have a variety of effects on the host, both local and systemic. Predominant among these toxins is pertussis toxin (PT), a multi-subunit protein toxin that inhibits signaling through a subset of G protein-coupled receptors in mammalian cells. PT activity has been linked with severe and lethal pertussis disease in young infants and a detoxified version of PT is a common component of all licensed acellular pertussis vaccines. The role of PT in typical pertussis disease in other individuals is less clear, but significant evidence supporting its contribution to pathogenesis has been accumulated from animal model studies. In this review we discuss the evidence indicating a role for PT in pertussis disease, focusing on its contribution to severe pertussis in infants, modulation of immune and inflammatory responses to infection, and the characteristic paroxysmal cough of pertussis.

## 1. Introduction

Pertussis toxin (PT) is an important virulence factor of the respiratory pathogen *Bordetella pertussis* [1]. PT is an adenosine diphosphate (ADP)-ribosylating protein toxin that inhibits signaling through a subset of G protein-coupled receptors (GPCRs) in mammalian cells. Pertussis has long been considered a toxin-mediated disease, with PT promoting the majority of pathogenic manifestations [2]. Evidence from both human disease and animal models of *B. pertussis* infection strongly indicates that PT is a major contributor to severe pertussis disease. In this review, we consider the association of PT activity with three different (but possibly related) aspects of pertussis disease: Severe disease in young infants; immune and inflammatory responses; and paroxysmal cough.

## 2. Role of PT in Severe Infant Pertussis

In 1984, the contribution of PT to *B. pertussis*-induced lethality was first described [3]. Using an infant mouse pertussis model, it was found that a mutant strain of *B. pertussis,* deficient in PT secretion, had a significantly higher LD_50_ than the parental wild type strain [3]. This result was later confirmed, with that same mutant requiring a higher challenge dose to achieve 100% lethality [4], without displaying impaired colonization [5]. However, the mechanism by which PT promoted lethality in these studies was not examined.

In humans, the most severe disease caused by *B. pertussis* typically manifests in infants aged <3 months. In the U.S., pertussis results in hospitalization for over 40% of infected individuals of this age group [6] and of those hospitalized infants, one in 100 dies as a result of the infection [7]. Severe disease is associated with the manifestation of a number of clinical complications that are not typically observed with pertussis in individuals older than six months. Severe pertussis complications include pneumonia, apnea, leukocytosis, pulmonary hypertension, seizures and encephalopathy [8,9,10,11]. Infant immune responses differ from older individuals, with an emphasis on a disease tolerance strategy rather than a disease resistance strategy [12]. Whilst this can be a beneficial strategy in some cases, it is likely that the inappropriate or insufficient immune response of the infant provides an opportunity for enhanced *B. pertussis* colonization, which can lead to exacerbated PT-mediated pathologies [13].

Three clinical manifestations of pertussis, in infants where PT may act as a potentiator, are leukocytosis, pulmonary hypertension, and brain pathologies, and these and other potentially PT-related complications are discussed in the following subsections.

### 2.1. PT as a Potentiator of Leukocytosis

There is a strong association between the number of circulating white blood cells and pertussis severity, with high white blood cell count (leukocytosis) and rapid progression of leukocytosis both being associated with increased risk of mortality [8,10,11,14]. In addition to increasing total white blood cell number, *B. pertussis* specifically elevates the number of circulating lymphocytes in infants but not older individuals [15]. Concurrent with lymphocytosis, *B. pertussis* also affects lymphoid organs. Infants that succumb to *B. pertussis* display advanced cortical depletion in the thymus, severe lymphocyte depletion in lymph nodes and advanced depletion of white pulp in the spleen [16]. These data suggest that *B. pertussis* promotes the egress of lymphocytes from the lymphoid system to establish leukocytosis. Interestingly, it appears that these cells migrate directly from lymphoid organs to the blood and not via lymphatic ducts [17]. In addition, once in the blood, leukocytes become trapped due to an impaired ability to extravasate (reviewed in Reference [18]). These data are indicative of impaired mechanisms of cellular trafficking as a result of *B. pertussis* infection.

Prior to the isolation and characterization of PT, it was known that a heat-labile component of *B. pertussis* was sufficient to induce leukocytosis in animals when administered systemically [19,20]. In later work, a single moiety isolated from a *B. pertussis* culture supernatant was found to induce leukocytosis, with a predominance of lymphocytes, in a dose-dependent manner in mice when administered via intravenous injection [21]. This factor, then termed lymphocytosis-promoting factor, later became known as PT. These works facilitated the establishment of an entirely new field of research in *B. pertussis* biology. Owing to the isolation and characterization of PT, studies led by Ui and colleagues were able to determine the molecular mechanisms of action of PT [22,23] and, as a result, a new tool for the study of G protein-coupled receptor (GPCR) signaling was identified.

Administration of purified PT induces leukocytosis in rats [24], pigs [25], and macaques [26,27]. Lymph node homing markers, such as the adhesion integrin leukocyte function antigen-1 (LFA-1), mediate lymphocyte attachment and extravasation at lymph node venules [28]. Studies using the macaque model determined that PT contributed to leukocytosis by reducing surface expression of LFA-1 on lymphocytes [29], creating a situation where lymphocytes fail to traffic effectively and get trapped in the vasculature. In addition, analysis of leukocytes from human infants with pertussis showed a marked reduction in surface expression of the lymph node homing marker L-selectin [30,31], further supporting the idea that PT traps leukocytes in the vasculature.

In those early studies described above, PT was administered directly to the animals systemically, but what is the evidence for PT-mediated induction of leukocytosis during *B. pertussis* infection? There is no direct evidence linking PT with *B. pertussis*-induced leukocytosis in humans, however, there are several lines of indirect evidence. There are two reports of natural *B. pertussis* infection of an infant by a strain deficient in PT expression [32,33]. In one report, the infant was three months of age and unvaccinated, and hence likely to develop leukocytosis upon *B. pertussis* infection. Consistent with a role for PT in promoting this pathology, leukocytosis did not develop in this infant [32]. In a second report, an unvaccinated 11-month-old did not develop pertussis-associated complications, but a white blood cell count was not discussed [33]. Human challenge studies with PT-expressing or isogenic PT-deficient strains of *B. pertussis* to determine a role for PT in leukocytosis have not been reported. In addition, given that *B. pertussis* only induces leukocytosis in infants and young children, such studies are unlikely to be performed. Studies on a live attenuated vaccine strain of *B. pertussis* may have the most potential to determine the function of PT in humans during *B. pertussis* infection. BPZE1 is a genetically modified *B. pertussis* strain developed as a nasally inoculated live vaccine that expresses a genetically detoxified version of PT [34]. Human studies on this vaccine candidate have thus far been limited to phase I clinical trials in adults to determine vaccine safety [35]. However, four- to five-month-old baboons inoculated with BPZE1 did not significantly increase white blood cell counts up to 42 days post-inoculation [36], whereas baboons aged up to nine months develop robust leukocytosis when infected with a recent clinical isolate of *B. pertussis* [37]. However, BPZE1 is also altered in its expression of dermonecrotic toxin and tracheal cytotoxin [34] and so one should be cautious when interpreting results in the presence of these other attenuations that could alter the pathogenesis of the disease. Humans are also a natural host for a related *Bordetella* species, *B. parapertussis*, which does not express PT. Infection with *B. parapertussis* does not result in leukocytosis [38]. However, *B. parapertussis* is not simply *B. pertussis* without PT; this species has a number of other genetic differences. Consequently, the factors contributing to the different clinical manifestations cannot easily be determined.

Another approach to determine the contribution of PT to leukocytosis during *B. pertussis* infection is to examine the effects of PT-targeted therapies on the course of the disease. There is currently no clinically approved PT-targeting agent and so large cohort studies demonstrating efficacy in humans have not yet been performed. However, the efficacy of monoclonal and polyclonal antibodies to PT has been explored extensively in animal models. Studies by Sato, Manclark and colleagues demonstrated that polyclonal and monoclonal antibodies to PT administered prior to *B. pertussis* challenge of mice were able to limit leukocytosis and concomitantly pertussis-induced mortality [39,40,41,42]. The therapeutic benefit of anti-PT antibodies administered after infection in mice has also been described [42,43]. In a small study examining the safety and pharmacology of intravenous pertussis immunoglobulin (P-IGIV) in *B. pertussis*-infected children <2 years of age, P-IGIV significantly reduced the number of circulating white blood cells after a single infusion [44]. More recently, the Maynard group has sought to develop an improved, humanized version of the monoclonal anti-PT antibody. Work by this group has demonstrated that humanized monoclonal PT-targeted antibodies constructed from efficacious murine antibody clones 1B7 and 11E6 [42] were able to mitigate *B. pertussis*-induced leukocytosis in baboons when administered prophylactically and at therapeutic time points [45]. It will be very interesting to see the efficacy of these and other potential therapies in a clinical setting and to explore how such PT-specific targeting alters the course of the disease.

### 2.2. PT as a Potentiator of Pulmonary Hypertension

Pulmonary hypertension associated with *B. pertussis* infection in infants is strongly correlated with disease severity, with 75% of infants that succumb to infection displaying features of pulmonary hypertension compared with just 6% of those that survive infection [8]. This intractable pathology is a known risk factor for death in infants with pertussis [11] and associated cardiac failure is often listed as a cause of death [9,46,47]. Understanding the cause of *B. pertussis*-induced pulmonary hypertension and the development of targeted therapeutics could save the lives of infected infants. Unfortunately, how *B. pertussis* induces pulmonary hypertension remains unknown. Reports have described the identification of leukocyte thrombi in small pulmonary blood vessels and speculated that vascular occlusion caused by leukocytosis results in increased pulmonary arterial pressure and establishment of pulmonary hypertension [48,49]. However, this finding is not supported in other clinicopathological studies [16,50]. If leukocytosis does promote the onset of pulmonary hypertension, this may be one mechanism by which PT indirectly promotes this pathology. Indeed, the Carbonetti group has found that infant mice infected with a PT-expressing strain of *B. pertussis* displayed features of pulmonary hypertension, while mice infected with a PT-deficient strain did not (Scanlon K., unpublished data). It is also hypothesized that PT could promote pulmonary hypertension by inhibiting signaling through age-related GPCRs in the heart and lungs [10]. One such GPCR could be the angiotensin II receptor AT_2_. AT_2_ receptors are highly expressed in fetal tissues, including the aorta [51,52], and expression then changes to a more balanced AT_1_:AT_2_ ratio in young children [53]. AT_2_, but not AT_1_, is a PT-sensitive GPCR that propagates the anti-proliferative, vasodilatory functions of angiotensin II [54,55,56]. During development, AT_2_ is thought to function by limiting excessive vascular smooth muscle cell proliferation [57], hence disruption of signaling through this receptor by PT could promote arterial thickening and vasoconstriction in an age-related manner, pathologies that would contribute to the development of pulmonary hypertension.

### 2.3. PT as a Potentiator of Brain Dysfunction

Infants with severe pertussis can manifest a number of brain pathologies, including seizure, encephalopathy, and hemorrhage [8,10,58,59]. Seizure or convulsions typically occur after cyanotic episodes and are thought to result from anoxia [60]. However, most forms of brain pathology are associated with disruption of the integrity of the blood-brain barrier [61]. In vitro studies have shown that PT permeabilizes human brain-derived microvascular cells [62] and enhances transmigration of macrophages and monocytes across a human brain microvascular endothelial layer. These findings suggest that if PT were to disseminate systemically and intoxicate endothelial cells in the brain, this would result in significant disruption of barrier integrity. Hence, it is possible that PT may function to exacerbate encephalopathies and neurological disorders by disrupting the blood-brain barrier.

### 2.4. Other Complications and the Role of PT

Pneumonia and apnea have not yet been directly associated with PT-mediated effects. Indeed, in regards to pneumonia, an experiment in which infant mice were infected with a PT-expressing or PT-deficient strain of *B. pertussis* demonstrated that it was the absence of PT that was associated with greatest airway inflammation [63]. However, while PT may not be the cause of pneumonia, it is possible that pneumonia may exacerbate the effect of PT. Pneumonia-associated disruption of the pulmonary vascular barrier may facilitate systemic dissemination of *B. pertussis* or proteins produced by the bacterium (including PT) to induce severe PT-mediated pathologies beyond the airways. In line with this, infants that display pertussis-induced pneumonia at an early stage of infection are more likely to develop severe disease, characterized by the development of pulmonary hypertension or death [10]. In addition, while infant mice infected with a PT-deficient strain of *B. pertussis* develop enhanced airway inflammation, these animals do not succumb to infection, a phenotype only observed with PT-expressing *B. pertussis* [63]. Therefore, whether pneumonia is a direct cause of death or a mechanism promoting systemic PT intoxication warrants further examination. Apnea may be spontaneous or, as is typical, occur after consecutive bouts of coughing [60,64]. The cause of apnea remains to be determined, but one hypothesis is that it is caused by alveolar atelectasis [60]. Alveolar collapse can result from fluid accumulation in the airways. Therefore, while PT has not been directly linked with apnea, its ability to promote lung edema may be a contributing factor [65,66]. In addition, PT may be promoting cough-induced perturbations to respiration rate by altering cough dynamics (see Section 4).

## 3. PT Effects on Inflammatory and Immune Responses to *B. pertussis* Infection

**Figure 1:**
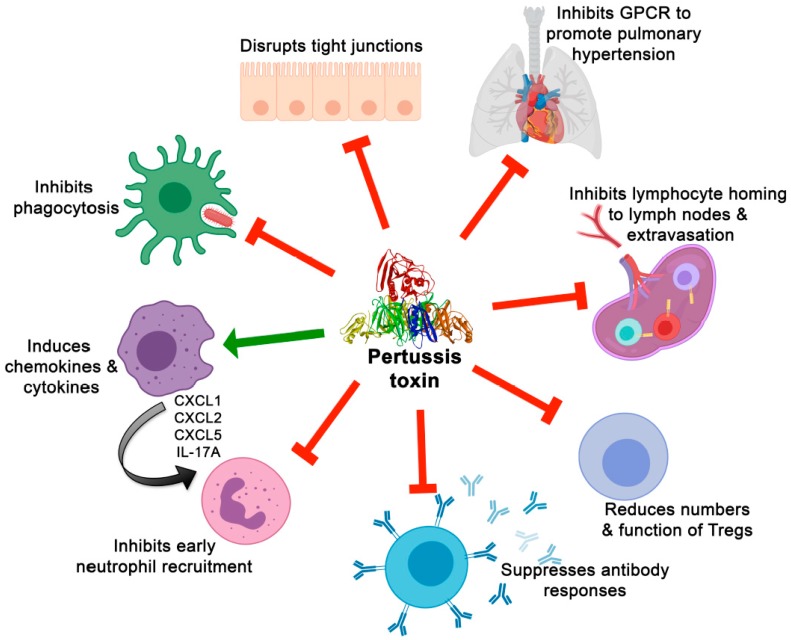
Effects of PT on immune cells and responses and on other aspects of pertussis pathogenesis.

Another function of PT that may contribute to disease pathogenesis is its effects on inflammatory and immune responses to *B. pertussis* infection. Figure 1 summarizes some of the effects of PT on immune cells and responses, as well as selected effects on pathogenesis. Animal models have played an important part in elucidating these effects, as well as advancing our understanding of *B. pertussis* pathogenesis and virulence mechanisms in general. Mice, rats, rabbits, non-human primates (NHPs), and pigs are among the species to have been utilized in the study of pertussis pathogenesis and vaccine development [37,67,68,69,70]. The majority of our understanding of PT modulation of host immune responses has come from studies involving the mouse and NHP models of disease. Most of these studies have used adult mice, in which immune responses differ from infant mice as discussed above.

### 3.1. Effects on Innate Immunity and Inflammation

*B. pertussis* attaches to ciliated cells of the respiratory tract and elicits robust cellular and humoral immune responses, the nature and kinetics of which have been reviewed previously [71]. These ciliated airway epithelial cells represent one of the earliest opportunities for innate defenses against the bacteria. PT-sensitive Gα subunits mediating GPCR signaling are involved in the biogenesis and maintenance of epithelial cell tight junctions [72]. PT, through inhibition of these GPCRs, is known to induce pulmonary edema in infected mice [66]. Alveolar macrophages (AM) are the first myeloid immune cells to interact with the pathogen and tasked with controlling infection. Clodronate liposome depletion of AM during *B. pertussis* infection in mice demonstrated that these cells are early targets for PT intoxication, which promotes *B. pertussis* survival in the respiratory tract [73]. Further, studies using human peripheral blood monocytes demonstrated the ability of PT to reduce monocyte phagocytosis [74].

Following AM defense, gamma-delta T cells, IL-17-producing macrophages, neutrophils, and IFN-γ+ NK cells are recruited during the bacteriostatic stage of infection [71]. Murine studies have shown that neutrophils are of little benefit during infection of naïve mice but are essential for control of infection in immune mice [75]. During the early stages of infection in naïve mice, PT mediates the inhibition of neutrophil recruitment to the lungs and airways [76]. By day four post-inoculation, however, PT promotes increased expression of neutrophil-attracting chemokines CXCL1, CXCL2, CXCL5, and IL-17A by macrophages, resulting in exacerbated neutrophil recruitment [77]. Treating mice with antibodies specific for IL-17A restored neutrophil recruitment to normal levels but this did not significantly impact the bacterial burden [77].

### 3.2. Effects on Adaptive Immunity

Clearance of *B. pertussis* coincides with the activation of neutrophils and macrophages in addition to adaptive IFN-γ- and IL-17-producing T cells [71]. Following this, the generation of immunoglobulin (Ig)G- and IgA-secreting plasma cells and central and effector memory T cells is required for immunity [71,78,79,80]. PT suppresses the ability of the host to generate effective antibody responses against *B. pertussis* antigens [81] despite its reported adjuvant activity [82]. Antibodies are necessary for control of infection in a mouse model [83] through their opsonization of bacteria for neutrophil killing and PT inhibits this process through its actions on neutrophil trafficking and recruitment [84]. PT suppression of antibody responses is supported by examples in the literature of the inhibitory effects of PT on B cell responses to lipopolysaccharide (LPS) [85], lymphocyte chemotaxis [86] and IgM production [87].

### 3.3. Effects on Resolution of Inflammation

Once the host has successfully contained *B. pertussis* infection through pro-inflammatory responses, an anti-inflammatory resolution phase is necessary to dampen and resolve the inflammatory response and prevent immune-mediated damage. Regulatory T cells (Tregs) play an important role in the resolution of inflammation [88] but the administration of purified PT to mice resulted in reduced frequency and immunosuppressive capabilities of splenic CD4+CD25+ Tregs [89]. This represents one of several potential mechanisms by which PT may inhibit resolution of pertussis disease. Several PT-sensitive GPCRs have been identified as having immune-resolving capabilities, such as sphingosine-1-phosphate receptor 1 (S1P1) [90], resolvin and lipoxin receptors [91], and cannabinoid receptor 2 [92]. PT has been shown to exacerbate and prolong the duration of respiratory inflammation in *B. pertussis*-infected adult mice [93], which we hypothesize is due to inhibition of pro-resolving signaling through these receptors. This highlights the dual nature of PT immune modulation in adult mice; initially suppressing the inflammatory response to enhance colonization, followed by an exacerbation of responses to promote pathogenesis. Drug targeting of one of these receptors, S1P1, has shown potential as a post-exposure therapeutic in the mouse model of *B. pertussis* infection [94,95].

## 4. Role of PT in Pertussis Cough

Although PT is considered a major virulence factor for *B. pertussis* and is clearly linked to leukocytosis and other aspects of pertussis disease pathology as described above, an important question is whether PT contributes to the severe cough manifestation of pertussis. While it is difficult to obtain definitive evidence from human disease studies, several lines of evidence indicate that PT is a major contributor to pertussis cough, as outlined below.

### 4.1. Evidence from Humans

Recent experimental infection of human volunteers with *B. pertussis* has been limited to nasal colonization with wild type [96] or candidate attenuated vaccine [97] strains, with no cough symptoms elicited. Since we cannot experimentally infect humans with isogenic wild type and PT-deficient *B. pertussis* strains to assess cough symptomology, evidence from humans indicating a role for PT in pertussis cough is more indirect. For example, an association between naturally occurring PT-deficient *B. pertussis* strains and reduced severity of cough symptoms in human pertussis cases would support a role for PT, but such strains are extremely rare and have only been reported in the published literature twice [32,33], as mentioned in Section 2. However, in neither case were the characteristics of cough reported. In addition, serology was not performed to assess the presence of anti-PT antibodies in the infected individuals and so it is impossible to know whether these strains lacked PT production at the onset of infection or whether a mutation occurred during infection or isolation and culturing of the strains. Indeed, recent studies show that multiple *B. pertussis* genetic variants can be isolated from single individuals with pertussis (Weigand, M.R., Williams, M.M., unpublished data), highlighting the plausibility of this scenario.

Another line of indirect evidence supporting a role for PT in pertussis cough disease can be derived from observations on human vaccination against pertussis. PT (detoxified by formaldehyde and/or glutaraldehyde) is a component of all currently used acellular pertussis vaccines, which typically include a small number of additional *B. pertussis* components [98]. However, one country, Denmark, has used a monocomponent PT vaccine (detoxified by hydrogen peroxide treatment) since the 1990s [99]. This vaccine was found to have the same range of efficacy against World Health Organization (WHO)-defined pertussis (including the cough duration) as the multicomponent acellular pertussis vaccines used in other countries [99,100]. Furthermore, the epidemiology of pertussis in Denmark is not significantly worse than that in other countries using multicomponent acellular pertussis vaccines, with inter-epidemic incidence below 10 per 100,000 and no significant epidemics since 2002 [99,101]. Since immune responses to the single PT component in this vaccine can effectively protect a population from pertussis disease, this supports the idea that PT is an important factor promoting pertussis cough disease. Additionally, the lack of identification of PT-deficient *B. pertussis* strains that would likely escape the vaccine-elicited immunity in this population argues for the overall importance of PT in pertussis infection and disease.

### 4.2. Evidence from Animal Models of Pertussis

Various animal models have provided direct and indirect evidence supporting a role for PT in the development and characteristics of pertussis cough. Although the existence of an as yet unidentified “cough toxin” produced by *B. pertussis* has been postulated [102], we propose that PT is the *B. pertussis* factor with the most significant contribution to pertussis cough. Direct evidence supporting this contention has been obtained from two separate animal model studies. In one, rats experimentally infected with *B. pertussis* developed a long-lasting paroxysmal cough [68], whereas rats equivalently infected with a PT-deficient strain (or with *B. parapertussis*, which does not produce PT) did not develop cough [103], indicating a role for PT in pertussis cough pathology. Similarly, young baboons, which develop prolonged paroxysmal coughing when experimentally infected with *B. pertussis* [37,104], did not develop cough or other pertussis disease symptoms when infected with an isogenic PT-deficient *B. pertussis* strain, despite being colonized similarly to animals infected with the PT-producing wild type strain (Merkel T., unpublished studies). In addition, similar to the indirect evidence from pertussis vaccination in humans, a recent study found that infant baboons born to mothers vaccinated with a monocomponent PT vaccine developed no cough or other disease symptoms, despite being heavily colonized after *B. pertussis* challenge [105]. This indicates that neutralization of PT activity can prevent the cough symptomology of pertussis, providing further support for a link between PT and pertussis cough.

But how is PT, a toxin that inhibits GPCR signaling, mechanistically linked to development and severity of cough in pertussis? It is apparently not a direct effect, since the administration of purified PT to humans or experimental animals does not elicit cough [106,107]. We propose that PT modulation of host responses to *B. pertussis* infection leads to both the development of cough and exacerbation of cough responses, resulting in the characteristic prolonged cough paroxysms associated with pertussis. As described above, we have found that PT activity is associated with prolonged and exacerbated respiratory inflammatory pathology in mouse models of *B. pertussis* infection [93,108]. This inflammatory pathology may be a prerequisite for cough development, thus explaining the lack of cough in rats and baboons infected with PT-deficient strains. Unfortunately, mice lack the ability to cough, which precludes direct testing of these hypotheses in mouse models. However, treatment of *B. pertussis*-infected mice with drugs that target S1P receptors dramatically reduces the level of lung inflammatory pathology [94,95,109] and one of these drugs is currently being tested in the baboon model of pertussis for its effect on inflammation and cough. In addition, mice infected with a PT-producing *B. pertussis* strain or treated with purified PT exhibit significantly exacerbated respiratory reflex responses (respiratory pauses and augmented breaths) to intratracheal administration of bradykinin, a cough stimulant and mediator of inflammation [106] (Canning and Carbonetti, unpublished data). This effect may be related to previously observed inhibition by PT of bradykinin receptor desensitization in in vitro studies [110,111], possibly explaining the prolonged cough paroxysms of pertussis. Since various GPCRs contribute to the regulation of cough responses [112], this effect of PT may extend beyond bradykinin receptors and inhibition by PT of receptor desensitization may prevent the cessation of the coughing response. Furthermore, our studies in mouse models have demonstrated that the effects of PT in the respiratory tract are long-lived [73,113], possibly explaining the protracted duration of pertussis cough paroxysms in individuals who have likely cleared the infection. Altogether, these lines of evidence strongly indicate a link between PT activity and cough responses in pertussis.

## 5. Conclusions

PT is a central factor in promoting disease pathogenesis in pertussis. In severe infant pertussis, leukocytosis and pulmonary hypertension are associated with PT-mediated dysfunction in organs outside the airways, suggesting that, in infants, either *B. pertussis* itself or its toxins are capable of disseminating beyond the primary site of infection. Animal models indicate an important role for PT in modulating many inflammatory and immune responses, an effect that, along with other physiological effects of PT, may contribute to the cough pathology. Therefore, therapeutics directed specifically at PT activity may be beneficial at reducing severe pertussis in infants and cough symptomology in other individuals with pertussis. Although treatment of children suffering from pertussis with P-IGIV produced inconclusive effects on cough [44,114], the recently developed humanized monoclonal antibodies to PT show beneficial effects in *B. pertussis*-infected experimental mice and baboons [45] and warrant further investigation. In addition, a recent study describes the inhibitory activity of cyclophilin inhibitors, such as cyclosporine A, on PT activity in cultured cells [115], potentially providing another avenue for the development of PT-directed therapeutics.
